# Validation of the Quantitative Checklist for Autism in Toddlers in an Italian Clinical Sample of Young Children With Autism and Other Developmental Disorders

**DOI:** 10.3389/fpsyt.2019.00488

**Published:** 2019-07-17

**Authors:** Liliana Ruta, Flavia Chiarotti, Giuseppe Maurizio Arduino, Fabio Apicella, Elisa Leonardi, Roberta Maggio, Cristina Carrozza, Natasha Chericoni, Valeria Costanzo, Nazarena Turco, Gennaro Tartarisco, Antonella Gagliano, Carrie Allison, Simon Baron Cohen, Giovanni Pioggia, Filippo Muratori

**Affiliations:** ^1^Institute for Biomedical Research and Innovation, National Research Council of Italy, Messina, Italy; ^2^Department of Developmental Neuroscience, Stella Maris Scientific Institute, Pisa, Italy; ^3^Center for Behavioral Sciences and Mental Health, National Institute of Health, Rome, Italy; ^4^Centro Autismo e Sindrome di Asperger ASLCN1, Mondovì, Italy; ^5^University of Messina, Messina, Italy; ^6^Autism Research Centre, Department of Psychiatry, University of Cambridge, Cambridge, United Kingdom; ^7^University of Pisa, Pisa, Italy

**Keywords:** autism, early screening, toddlers, Quantitative Checklist for Autism in Toddlers, Autism Spectrum Disorders (ASD)

## Abstract

**Background:** The Quantitative Checklist for Autism in Toddlers (Q-CHAT) is parent-report screening questionnaire for detecting threshold and sub-threshold autistic features in toddlers. The Q-CHAT is a dimensional measure normally distributed in the general population sample and is able to differentiate between a group of children with a diagnosis of autism and unselected toddlers.

**Objectives:** We aim to investigate the psychometric properties, score distribution, and external validity of the Q-CHAT in an Italian clinical sample of young children with autism versus children with developmental delay and typically developing children.

**Method:** N = 126 typically developing children (TD), n = 139 children with autism, and n = 50 children presenting developmental delay (DD) were administered the Q-CHAT. Standardized measures of cognitive functions, language, and behaviors were also obtained.

**Results:** The Q-CHAT scores were normally distributed and demonstrated adequate internal consistency and good item to total score correlations. The mean Q-CHAT score in the autism group was significantly higher than those found in the DD sample and TD children. No difference on the mean Q-CHAT score between DD and TD children was found. The accuracy of the Q-CHAT to discriminate between autism and TD was very good. Two different cut-points (27 and 31, respectively) maximized sensitivity and specificity for autism versus TD and DD, respectively. Finally, higher Q-CHAT scores were correlated with lower language and social communication skills.

**Conclusions:** In clinical settings, the Q-CHAT demonstrated good psychometric properties and external validity to discriminate autism children not just from children with typical development but also from children with developmental delay.

## Introduction

Autism spectrum conditions (autism) are a neurodevelopmental condition that significantly impairs social communication and includes unusually narrow interests and difficulties adjusting to unexpected change (1). Autism begins very early in life and is lifelong. There is consolidated evidence that early intervention has a significant impact on reducing the severity of symptoms and improving social communicative and adaptive skills with consequent better functioning and greater independence later in life (2, 3). However, early intervention is possible only if children at risk can be detected accurately through autism-specific screenings by the age of 18–24 months and immediately referred for diagnostic assessment. For these reasons, the American Academy of Pediatrics (4) and the Centers for Disease Control and Prevention’s National Center on Birth Defects and Developmental Disabilities (NCBDDD) (5) have recommended the use of routine screeners within developmental surveillance to help pediatricians develop a strategy for early identification of children with autism. Different screening instruments for autism, with different scoring approaches (categorical versus continuous), have been developed since the late 1990s and used as first-level screeners in community samples and/or as level 2 screeners in clinical settings (6–8). Among them, the Checklist for Autism in Toddlers (CHAT) (9), the Modified Checklist for Autism in Toddlers (M-CHAT) (10), and the M-CHAT-Revised with Follow-up (11) (M-CHAT/RF) have been tested in the general population. Results indicated that the CHAT at 18 months had a high specificity and positive predictive value but low sensitivity, dropping too many affected children. The M-CHAT and M-CHAT/RF, which replaced the CHAT, have been validated across multiple studies, cultures, and populations, mostly in mixed samples of high- and low-risk children and have demonstrated moderate psychometric properties (12, 13). In high-risk samples of children referred for developmental concerns, as expected considering the higher prevalence of autism, the M-CHAT demonstrated higher positive predictive values (PPVs) of 0.74 (14) and 0.79 (15), respectively, in two independent samples. Similar PPVs were also reported for other screeners such as the Social Communication Questionnaire (SCQ) (PPV of 65%) (16) and the ESAT (PPV of 79%) (17). Other studies, conducted in clinical settings, compared the score distribution and accuracy of different screeners in young children with a diagnosis of autism, children with other developmental conditions, and typically developing children. Stone et al. (18) reported that scores on the Screening Tool for Autism in Two-Year-Olds (STAT) in children with autism were significantly higher than those reported in children with developmental delay and/or language impairment. Similarly, Matson et al. (19) tested the validity of the Baby and Infant Screen for Children with aUtIsm Traits (BISCUIT) to identify autism in a cohort of children presenting either developmental delay and/or medical conditions likely to result in a developmental delay. BISCUIT-Part 1 total scores in the autism group were significantly higher than those reported in the control group with developmental conditions. In another preliminary study on the Quantitative Checklist for Autism in Toddlers (Q-CHAT), Allison et al. (20) examined the clinical validity of the Q-CHAT as a dimensional measure of threshold and sub-threshold autistic features and found that the Q-CHAT was normally distributed in the general population sample and was able to differentiate between a group of children with a diagnosis of autism and unselected toddlers. In a subsequent study (21), a short version of the Q-CHAT (QCHAT-10), including the 10 items that best differentiated between children with and without autism, was tested and the screening cut-point of 3 demonstrated sensitivity and specificity estimates as high as 91% and 89%, respectively. Although the Q-CHAT results were promising, the full range of psychometric characteristics was not reported and the accuracy of the instrument with regard to other developmental conditions was not explored.

The current study aims to further investigate the Q-CHAT validity and score distribution in an independent clinical sample of young children with a diagnosis of autism, children with a diagnosis of developmental delay, and typically developing children. We also analyzed the accuracy of the Q-CHAT total scores in predicting diagnostic status in children with both autism and developmental delay. Finally, we explored the predictive validity of screening scores on the Q-CHAT with regard to measures of cognitive functioning, language, behaviors, and autism symptom severity.

## Methods

### Participants

A group of n = 315 young children [M/F = 206:109 (65%:35%), mean age (SD) = 31.6 (8.8) months] from three Italian regions (Piedmont, Tuscany, and Sicily) took part in the study. N = 126 were typically developing children (TD) [mean age (SD) = 33.2 (9.3) months], n = 139 children had a diagnosis of autism [mean age (SD) = 31.6 (8.0) months], and n = 50 children were presenting Developmental Delay (DD) [mean age (SD) = 27.6 (8.3) months]. TD children were recruited in mainstream nursery schools. Parents were given the QCHAT through the teachers, and the completed questionnaires were collected back by a member of the research team at school. Autism and DD children were diagnosed and tested at the clinical facilities within the Autism Centre (C.A.S.A.) of the NHS Unit CN1 in the province of Cuneo (Piedmont), the Scientific Foundation “Stella Maris” in Pisa (Tuscany), and the University Hospital “G. Martino” in Messina (Sicily). Parents were given the QCHAT by a member of the research team and filled out the questionnaire during the child’s assessment. All parents were explicitly asked to fill out the questionnaire together.

### Procedure

The study was conducted as part of a large population-based screening program funded by the Ministry of Health and Tuscany Region (GR-2010-2319668). The study was approved by the local Ethic Committees in each region, and all the participants signed a written consent form to be enrolled in the study. All the participants, including TD children, were given the Griffith’s Mental Development Scale (22) to assess their language and performance developmental quotient (LDQ and PDQ). TD children presenting either language or global developmental delay (n = 2) as well as autistic traits (n = 1) were excluded from the study and offered a separate dedicated diagnostic assessment. The Autism Diagnostic Observation Schedule, Second Edition (ADOS-2) (23) was used as part of the diagnostic assessment in the autism group. DD and autism diagnoses were made by multidisciplinary teams comprising psychologists and child neuropsychiatrists according to DSM 5 criteria of ASD and global developmental delay. Furthermore, parents of autism and TD children completed the Child Behavior Checklist 1.5-5 (24).

### Validation of the Italian Q-CHAT

The Q-CHAT is a 25-item caregiver-report screening measure for autistic traits in toddlers. Items are rated on a five-point Likert scale (0–4), with higher ratings indicating more autistic traits and a Q-CHAT total score ranging from 0 to 100. Thirteen items are reverse scored. The scoring procedure used in the study was exactly the same as that used in the original Q-CHAT study by Allison et al. (20). To maintain the functional and conceptual equivalence of words and sentences between English and Italian, a back-translation was conducted and points of divergence were discussed with the authors who developed the instrument (CA and SBC) to ensure that the items were accurately reflecting the same meaning as that in the original language.

### Statistical Analyses

All statistical analyses were conducted using the Data Analysis and Statistical Software STATA Release 8.1 (25). As per Allison et al. (20), incomplete or ambiguously answered Q-CHAT items were conservatively scored “0.” If seven or more Q-CHAT items were missing, then the checklist was excluded from analysis [n (%) = 3 (0.9%)]. Accordingly, for the CBCL, missing items were conservatively scored as “0,” whilst questionnaires with more than eight missing items were excluded [n (%) = 3 (1.1%)] (24). Descriptive analysis was conducted on personal history as well as socio-demographic status, accounting for group, gender, and region. In particular, categorical variables were analyzed using the chi-squared test, while quantitative variables were analyzed using either the Student t test or the analysis of variance (ANOVA). Cramer’s V and eta-squared were computed as measures of effect size for categorical and quantitative variables, respectively. Multiple comparisons were performed by applying Holm–Bonferroni’s correction to Fisher’s exact probability test and for categorical variables, and the Tukey test for quantitative variables. The Shapiro–Wilk test was used to assess normality in the Q-CHAT score distribution. Q-CHAT item distribution and item–total correlations were also examined using Spearman’s rho non-parametric correlation coefficient in each group separately. Cronbach’s alphas were calculated to examine the Q-CHAT total score internal consistency in each group and the overall sample. A between-group analysis of covariance, accounting for the effect of age and PDQ, was conducted to assess group differences in the Q-CHAT total scores. In addition, a multiple linear regression model was applied to assess the effect of group, gender, age, Performance Developmental Quotient (PDQ), and parental education on QCHAT total scores. A receiver operating characteristic (ROC) curve of the Q-CHAT total score was produced to plot sensitivity and 1-specificity in relation to both an autism and DD diagnosis. The area under the curve (AUC) is a measure of the overall predictive validity, where an AUC = 0.50 indicates random prediction of the independent variable and an AUC > 0.90 indicates excellent validity. Potential cutoff scores on the Q-CHAT for differentiating between children with autism, DD, and TD were also evaluated using ROC analysis to determine the cut-point corresponding to the best combination of sensitivity and specificity. The relationship between the Q-CHAT scores LDQ and PDQ as well as the ADOS-2 scores in the ASD group was examined using a multiple linear regression model that accounted for the effects of age, gender, and parental education. Finally, convergent validity between the Q-CHAT total score and the CBCL 1.5-5 domains in autism and TD children separately was assessed using Spearman’s rho non-parametric correlation coefficient.

## Results

### Demographic and Clinical Characteristics of the Sample

**Table 1** reports the demographic and clinical characteristics of the sample.

**Table 1:** Descriptive and clinical characteristics of children with autism, developmental delay (DD), and typically developing children (TD).

	Autism	DD	TD	Group	Region	Group*region
N	139	50	126	/	/	/
Age in months (mean, SD)	31.6 (8)	27.6 (8.3)	33.2 (9.3)	F(2,297) = 4.8, p = 0.009	F(2,297) = 1.3, p = .3	F(4,297) = 4.4, p = 0.002^§^
Gender M:F (N, %)	116:23 (83:17)	29:21 (58:42)	59:67 (47:53)	X^2^(2) = 40.6, p < 0.001	X^2^(2) = 2.3, p = .3	X^2^(8) = 48, p < 0.001^§^
PDQ	88 (34.1)	61.8 (9.6)	119.6 (21.5)	F(2,282) = 67.7, p < 0.001	F(2,282) = 0.6, p = .6	F(4,282) = 0.98, p = .4
Q-CHAT total score*	39.4 (13.1)	27.1 (6.3)	21.1 (6.7)	F(2,272) = 72.6, p < 0.001	F(2,272) = 1.4, p = .2	F(4,272) = 1.2, p = .3
**Personal history**						
Term pregnancy (N, %)	116 (86)	35 (78)	104 (88)	X^2^(2) = 2.9, p = .2	X^2^(2) = 1.6, p = .4	X^2^(8) = 10.9, p = .2
Pregnancy complications (N, %)	22 (18)	4 (10)	10 (9.5)	X^2^(2) = 3.7, p = .2	X^2^(2) = 9.1, p = 0.01^§^	X^2^(8) = 15.9, p = 0.04^§^
Birth weight in gr. (Mean, SD)	3,280 (549.6)	2,876.9 (768.1)	3,170.1 (537.8)	F(2,287) = 1.7, p = .2	F(2,287) = 3.9, p = 0.02^§^	F(4,287) = 3, p = 0.02^§^
APGAR score (Mean, SD)	9 (0.7)	9.1 (1.1)	9.3 (0.7)	F(2,153) = 2.6, p = 0.08	F(2,153) = 1.5, p = .2	F(4,153) = 1, p = .4
Perinatal problems (N, %)	19 (15)	8 (19)	14 (12)	X^2^(2) = 1.4, p = .5	X^2^(2) = 11.6, p = 0.003^§^	X^2^(8) = 14.2, p = 0.07
Gait in months (Mean, SD)	14.1 (2.7)	18.2 (1.8)	12.7 (2.2)	F(2,256) = 42.2, p < 0.001	F(2,256) = 4.5, p = 0.01^§^	F(4,256) = 1.8, p = .1
First words in month (Mean, SD)	17.5 (8.7)	17 (2.3)	10.8 (3.6)	F(2,220) = 25.5, p < 0.001	F(2,220) = 0.2, p = .8	F(4,220) = 0.5, p = .7
Nursery school (N, %)	89 (66)	25 (50)	119 (95)	X^2^(2) = 50, p < 0.001	X^2^(2) = 4.5, p = .1	X^2^(8) = 66.8, p < 0.001^§^
**SES**						
Education mother (N, %)				X^2^(6) = 19.6, p = 0.01	X^2^(6) = 18.1, p = 0.02^§^	X^2^(16) = 38.9, p = 0.001^§^
Pre-primary, primary	24 (17)	12 (26)	7 (6)			
Secondary	57 (42)	18 (39)	53 (42)			
Bachelor, Master Degree, PhD	56 (41)	16 (35)	65 (52)			
Occupation mother (N, %)				X^2^(6) = 35.5, p < 0.001	X^2^(6) = 12.5, p = 0.05	X^2^(24) = 47.7, p = 0.003^§^
Not working	56 (41.5)	24 (52)	25 (20.5)			
Manual, technical	10 (7.5)	2 (4)	5 (4)			
Clerical, sales	37 (27)	16 (35)	34 (28)			
Administrative, professional, management	32 (24)	4 (9)	58 (47.5)			
Ethnicity mother				X^2^(6) = 11.5, p = 0.07	X^2^(6) = 10, p = .1	X^2^(24) = 40.5, p = 0.02^§^
Caucasian	133 (96.5)	43 (95.5)	123 (98.5)			
Asiatic	0	2 (4.5)	2 (1.5)			
African	2 (1.5)	0	0			
Other	3 (2)	0	0			
Education father				X^2^(6) = 8.3, p = .4	X^2^(6) = 15.5, p = 0.05	X^2^(16) = 30.9, p = 0.01^§^
Pre-primary, primary	34 (25)	15 (32.5)	23 (20)			
Secondary	59 (43)	29 (43.5)	51 (44)			
Bachelor, Master Degree, PhD	43 (32)	11 (24)	42 (36)			
Occupation father				X^2^(6) = 19.4, p = 0.004	X^2^(6) = 15, p = 0.02^§^	X^2^(24) = 41.8, p = 0.01^§^
Not working	4 (3)	7 (15.5)	5 (4)			
Manual, technical	47 (35)	15 (33.5)	25 (21)			
Clerical, sales	35 (26)	9 (20)	29 (25)			
Administrative, professional, management	49 (36)	14 (31)	59 (50)			
Ethnicity father				X^2^(6) = 5.3, p = .3	X^2^(6) = 11.2, p = 0.02^§^	X^2^(24) = 32.7, p = 0.01^§^
Caucasian	136 (98)	43 (96)	119 (99)			
Asiatic	0	1 (2)	1 (1)			
African	1 (2)	1 (2)	0			
Other	0	0	0			

*Controlled for age and PDQ; §p > 0.05 after Bonferroni-Holm correction.

Within each group, no regional differences were found for the main demographic and clinical characteristics of the sample (all p > 0.05 after Bonferroni–Holm correction). Furthermore, neither a main effect of region nor a region by group interaction was found for the Q-CHAT scores; hence, all the relevant analyses were conducted on the whole sample. As expected, a significant group difference in gender distribution was found (Chi squared = 40.61, df = 2, p < 0.001). The autism group had more males than females compared to the DD and TD groups (p < 0.001 for both comparisons), while no difference in gender distribution was found between DD and TD children. A significant difference between groups was also found for age [F(2,303) = 7.39, p < 0.001]. DD children in the sample were significantly younger than autism and TD children (p < 0.01 for both comparisons), while age between autism and TD children did not significantly differ. Furthermore, Performance Developmental Quotient (PDQ) scores were significantly different between the three groups [F(2,288) = 84.59, p < 0.001], with TD children having a significantly higher PDQ than ASD and DD children and autism children having in turn higher PDQ scores than DD children (p < 0.01 for each of the three pairwise comparisons).

### Q-CHAT Internal Consistency, Item Score Distribution, and Item–Total Correlations

The QCHAT scores were normally distributed in the ASD, DD, and TD groups (W = 0.98, p = 0.07, W = 0.97, p = 0.32, and W = 0.996, p = 0.97). Internal consistency was good in the overall sample as well as the autism group (Cronbach’s alpha = 0.87 and 0.84, respectively), and adequate in the DD and TD groups (Cronbach’s alpha = 0.70 for both). The item–score distribution of the Q-CHAT in the autism, DD, and TD groups is shown in **Table 2**.

**Table 2:** Item–score distribution in children with autism, developmental delay (DD), and typically developing children (TD).

	Autism					DD					TD				
	0	1	2	3	4	0	1	2	3	4	0	1	2	3	4
1. Look when call name	15.8	28.1	33.1	20.1	2.9	64.0	30.0	6.0	0.0	0.0	66.7	31.0	2.4	0.0	0.0
2. Eye contact	10.8	49.6	33.1	5.8	0.7	54.0	46.0	0.0	0.0	0.0	71.4	28.6	0.0	0.0	0.0
3. Lineup objects^a^	33.1	23.0	25.2	12.9	5.8	16.0	10.0	34.0	22.0	18.0	6.3	21.4	37.3	23.8	11.1
4. Understand child’s speech	5.0	10.8	21.6	13.7	48.9	32.0	26.0	24.0	6.0	12.0	33.3	41.3	19.0	6.3	0.0
5. Protoimperative pointing	41.7	24.5	5.0	5.0	23.7	66.0	18.0	8.0	2.0	6.0	53.2	27.0	11.9	4.8	3.2
6. Protodeclarative pointing	24.5	25.9	11.5	6.5	31.7	74.0	10.0	4.0	6.0	6.0	69.0	15.9	5.6	6.3	3.2
7. Interest in spinning object^a^	44.6	36.0	14.4	4.3	0.7	48.0	34.0	12.0	4.0	2.0	49.2	44.4	4.8	1.6	0.0
8. Number of words^a^	11.5	7.9	21.6	29.5	29.5	26.0	18.0	28.0	26.0	2.0	56.0	19.2	16.8	7.2	0.8
9. Pretend play	23.0	25.9	15.1	10.1	25.9	50.0	26.0	16.0	2.0	6.0	71.2	15.2	12.0	0.8	0.8
10. Follow a look	19.4	41.7	12.9	9.4	16.5	52.0	30.0	14.0	2.0	2.0	61.9	26.2	7.9	1.6	2.4
11. Sniff/lick unusual objects^a^	13.7	41.7	20.1	12.9	11.5	4.0	42.0	14.0	22.0	18.0	17.5	48.4	13.5	12.7	7.9
12. Use of hand as tool^a^	15.1	11.5	18.0	30.2	25.2	26.0	8.0	12.0	36.0	18.0	48.4	21.4	7.1	16.7	6.3
13. Walk on tiptoes^a^	27.3	30.9	24.5	12.9	4.3	40.0	24.0	30.0	6.0	0.0	50.8	23.0	19.8	6.3	0.0
14. Adapt to change in routine	42.4	42.4	9.4	3.6	2.2	38.0	56.0	6.0	0.0	0.0	40.5	50.8	7.1	1.6	0.0
15. Offer comfort	12.9	15.8	23.0	20.9	27.3	24.0	40.0	26.0	8.0	2.0	35.7	36.5	23.8	2.4	1.6
16. Does same thing over and over again^a^	19.4	17.3	22.3	25.9	15.1	20.0	10.0	14.0	24.0	32.0	27.8	24.6	19.8	17.5	10.3
17. Typicality of first words	34.8	24.6	9.4	1.4	29.7	62.0	24.0	6.0	2.0	6.0	73.8	23.8	1.6	0.8	0.0
18. Echolalia^a^	29.7	5.8	8.7	26.8	29.0	12.0	2.0	6.0	34.0	46.0	7.1	5.6	10.3	27.8	49.2
19. Gestures	31.7	28.8	8.6	11.5	19.4	68.0	28.0	4.0	0.0	0.0	70.4	24.8	4.0	0.8	0.0
20. Unusual finger movements^a^	68.3	9.4	4.3	12.2	5.8	76.0	6.0	10.0	6.0	2.0	88.9	4.0	4.8	0.8	1.6
21. Check reaction	17.3	28.8	29.5	15.8	8.6	34.0	38.0	24.0	4.0	0.0	32.8	41.6	20.8	4.8	0.0
22. Maintenance of interest^a^	49.3	34.1	9.4	5.8	1.4	48.0	32.0	18.0	2.0	0.0	52.0	29.6	16.0	2.4	0.0
23. Twiddle objects repetitively^a^	72.7	8.6	8.6	7.2	2.9	70.0	12.0	10.0	4.0	4.0	73.0	11.9	10.3	4.8	0.0
24. Oversensitive to noise^a^	43.2	20.1	23.7	12.2	0.7	50.0	30.0	14.0	6.0	0.0	45.2	32.5	18.3	2.4	1.6
25. Stare at nothing with no purpose^a^	56.1	18.7	8.6	12.9	3.6	86.0	6.0	4.0	4.0	0.0	90.5	7.9	1.6	0.0	0.0

a Reverse-scored items.

Most of the items were significantly correlated with the Q-CHAT total score in the overall group of children, with large effect sizes (0.50 ≤ *rho* ≤ 0.65) for items 1, 2, 4, 6, 8, 9, 10, 12, 15, 17, 19, and 25, moderate effect sizes (0.40 ≤ *rho* < 0.50) for items 16 and 20, and small effect sizes (0.20 ≤ *rho* < 0.40) for items 5, 7, 11, 13, 21, 23, and 24. Low effect sizes (*rho* < 0.20) were found for items 3, 14, 18, and 22.

### Group Differences in the Q-CHAT Scores

The mean Q-CHAT scores (SD) were 39.4 (13.1) in the autism group, 27.1 (6.3) in the DD sample, and 21.1 (6.7) for TD children.

**Figure 1:**
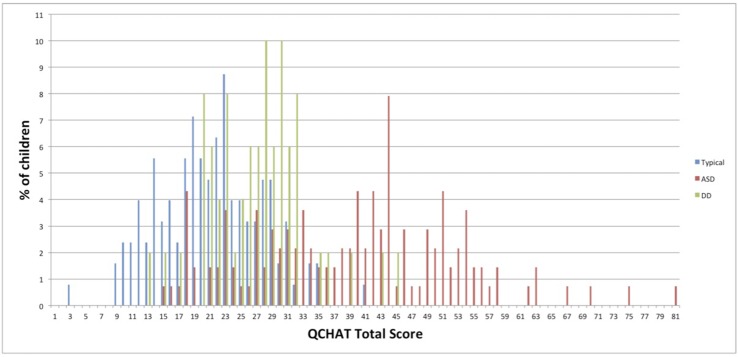
Q-CHAT score distribution in children with autism, developmental delay (DD), and typically developing children (TD).

**Figure 1** shows the Q-CHAT total score distribution in the three groups.

Since age and PDQ were significantly different between the three groups, an ANCOVA was performed to control for the effect of these variables. Adjusting for age and PDQ, a main effect of group on the QCHAT total scores was found [F(2,278) = 87.4, p < 0.001, eta squared = 0.46]. Pairwise comparisons indicated that Q-CHAT scores in the autism group were significantly higher than in the DD and TD groups (both p-values < 0.001). No difference in the Q-CHAT scores between DD and TD children was found (p = 0.56). When the effect of gender (controlled for age and PDQ) was explored, no main effect of gender [F(1,274) = 0.17, p = 0.68] nor gender by group interaction [F(2,274) = 0.24, p = 0.79] on Q-CHAT total score was found. Adjusted mean (SD) Q-CHAT scores by gender were as follows: autism males = 39.2 (10.4); autism females = 40.0 (10.0); DD males = 24.3 (10.6); DD females = 22.9 (10.7); TD males = 23.3 (10.7); TD females = 22.1 (10.8). In agreement with the ANCOVA, the multiple linear regression model including group, gender, age, PDQ, and parents’ education showed no significant effect on the Q-CHAT total score of gender (Beta = −0.27, p = 0.85), age (Beta = −0.10, p = 0.17), and the father’s education (medium- vs low-level: Beta = −1.90, p = 0.24; high- vs low-level: Beta = −3.01, p = 0.12). The mother’s medium-level and high-level education and PDQ were associated with lower QCHAT scores (education: Beta = −3.92 and −5.72, p = 0.05 and 0.01; PDQ: Beta = −0.06, p = 0.007). Finally, the QCHAT total score was markedly affected by the autism condition (Beta = 16.2, p < 0.001), but not by the DD condition (Beta = −0.2, p = 0.94) as compared to the TD condition.

### Accuracy of the Q-CHAT in Predicting ASD and Q-CHAT Cut-Points

**Figure 2:**
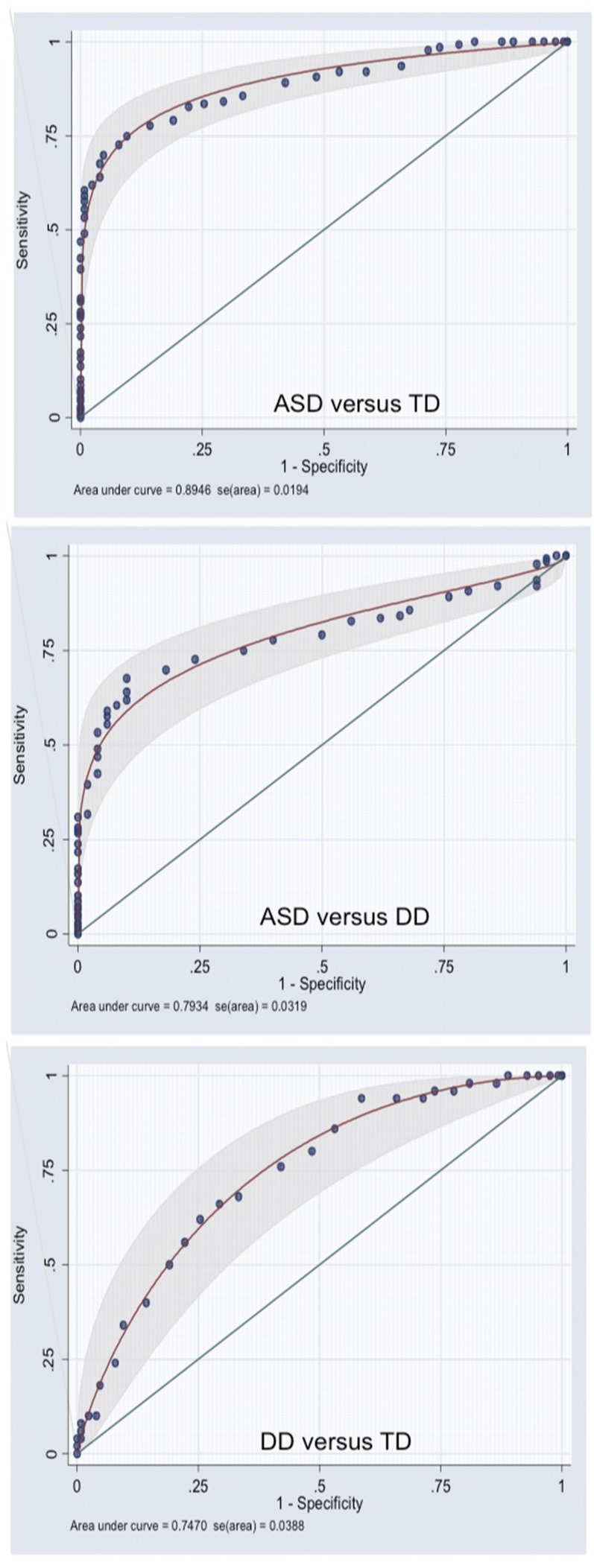
Area under the curve for the Q-CHAT.

**Figure 2** shows the area under the curve (AUC) for the Q-CHAT total score in the ASD versus TD, ASD versus DD, and DD versus TD groups.

Sensitivity and specificity associated with different cutoff scores for autism and DD children are presented in **Table 3**.

**Table 3:** Sensitivity and specificity of different Q-CHAT cut-points in predicting an autism and a DD status.

Cut-off	ASD versus TD		ASD versus DD		Cut-off	ASD versus TD		ASD versus DD	
	Sensitivity	Specificity	Sensitivity	Specificity		Sensitivity	Specificity	Sensitivity	Specificity
> = 9	100.00%	0.79%			> = 37	58.99%	99.21%	58.99%	94.00%
> = 10	100.00%	2.38%			> = 38	57.55%	99.21%	57.55%	94.00%
> = 11	100.00%	4.76%			> = 39	55.40%	99.21%	55.40%	94.00%
> = 12	100.00%	7.14%			> = 41	48.92%	99.21%	48.92%	96.00%
> = 13	100.00%	11.11%	100.00%	0.00%	> = 42	46.76%	100.00%	46.76%	96.00%
> = 14	100.00%	13.49%			> = 43	42.45%	100.00%	42.45%	96.00%
> = 15	100.00%	19.05%	100.00%	2.00%	> = 44	39.57%	100.00%	39.57%	98.00%
> = 16	99.28%	22.22%	99.28%	4.00%	> = 45	31.65%	100.00%	31.65%	98.00%
> = 17	98.56%	26.19%	98.56%	4.00%	> = 46	30.94%	100.00%	30.94%	100.00%
> = 18	97.84%	28.57%	97.84%	6.00%	> = 47	28.06%	100.00%	28.06%	100.00%
> = 19	93.53%	34.13%	93.53%	6.00%	> = 48	27.34%	100.00%	27.34%	100.00%
> = 20	92.09%	41.27%	92.09%	6.00%	> = 49	26.62%	100.00%	26.62%	100.00%
> = 21	92.09%	46.83%	92.09%	14.00%	> = 50	23.74%	100.00%	23.74%	100.00%
> = 22	90.65%	51.59%	90.65%	20.00%	> = 51	21.58%	100.00%	21.58%	100.00%
> = 23	89.21%	57.94%	89.21%	24.00%	> = 52	17.27%	100.00%	17.27%	100.00%
> = 24	85.61%	66.67%	85.61%	32.00%	> = 53	15.83%	100.00%	15.83%	100.00%
> = 25	84.17%	70.63%	84.17%	34.00%	> = 54	13.67%	100.00%	13.67%	100.00%
> = 26	83.45%	74.60%	83.45%	38.00%	> = 55	10.07%	100.00%	10.07%	100.00%
> = 27	82.73%	77.78%	82.73%	44.00%	> = 56	8.63%	100.00%	8.63%	100.00%
> = 28	79.14%	80.95%	79.14%	50.00%	> = 57	7.19%	100.00%	7.19%	100.00%
> = 29	77.70%	85.71%	77.70%	60.00%	> = 58	6.47%	100.00%	6.47%	100.00%
> = 30	74.82%	90.48%	74.82%	66.00%	> = 62	5.04%	100.00%	5.04%	100.00%
> = 31	72.66%	92.06%	72.66%	76.00%	> = 63	4.32%	100.00%	4.32%	100.00%
> = 32	69.78%	95.24%	69.78%	82.00%	> = 67	2.88%	100.00%	2.88%	100.00%
> = 33	67.63%	96.03%	67.63%	90.00%	> = 70	2.16%	100.00%	2.16%	100.00%
> = 34	64.03%	96.03%	64.03%	90.00%	> = 75	1.44%	100.00%	1.44%	100.00%
> = 35	61.87%	97.62%	61.87%	90.00%	> = 81	0.72%	100.00%	0.72%	100.00%
> = 36	60.43%	99.21%	60.43%	92.00%	> 81	0.00%	100.00%	0.00%	100.00%

In light gray is reported the best cut-point in predicting a DD status, in dark gray is reported the best cut-point in predicting an autism status.

Based on ROC analysis, the Q-CHAT total score that better differentiated between autism and TD children maximizing sensitivity (i.e., correctly identifying all children at risk for autism) while maintaining adequate specificity (i.e., correctly identifying all children not at risk for autism) was 27 (Sens. = 83%, Spec. = 78%). When autism children were compared to DD children, a higher cut-point of 31 or above indicative of an autism condition was found (Sens. = 73%, Spec. = 76%).

### Convergent Validity of the Q-CHAT With the Griffiths Development Quotient, the ADOS 2, and the CBCL.

In the autism sample, the QCHAT total scores were positively correlated with the ADOS 2 social affect (Beta = 0.94, p < 0.001) and negatively correlated with the Griffiths LDQ (Beta = −0.1, p = 0.02). No main effect of PDQ and ADOS 2 restricted and repetitive behaviors was found (Beta = 0.01, p = 0.72 and Beta = 0.13, p = 0.82). Furthermore, in both the autism and TD groups, the QCHAT total score was positively correlated with most of the CBCL domains with medium to large effect sizes in both groups (Spearman rho from 0.29 to 0.44 in autism and from 0.46 to 0.57 in TD children). The correlations between all the CBCL domains and the QCHAT scores in the autism and TD groups are reported separately in **Table 4**.

**Table 4:** Correlation of the QCHAT total score with the CBCL scores.

	Spearman rho		p-value	
	ASD	TD	ADS	TD
EMOTIONALLY REACTIVE	0.27	0.52	0.02	<0.001
ANXIOUS DEPRESSED	0.19	0.40	0.08	0.003
SOMATIC COMPLAINTS	0.003	0.45	0.98	0.001
WITHDRAWN	0.52	0.41	<0.001	0.003
SLEEP PROBLEMS	0.10	0.13	0.37	0.35
ATTENTION PROBLEMS	0.34	0.28	0.002	0.05
AGGRESSIVE BEHAVIOR	0.19	0.43	0.09	0.001
INTERNALIZING	0.38	0.57	<0.001	<0.001
EXTERNALIZING	0.29	0.46	0.01	0.001
PDD	0.44	0.49	<0.001	<0.001
ADHD	0.24	0.22	0.03	0.11
AFFECTIVE	0.23	0.27	0.04	0.05
ANXIETY	0.07	0.41	0.55	0.002
OPPOSITIONAL DEFIANT	0.26	0.34	0.02	0.01
TOTAL SCORE	0.34	0.55	0.002	<0.001

## Discussion

This study aimed to investigate the psychometric properties of the Q-CHAT among children with a diagnosis of autism and children presenting other neurodevelopmental conditions such as developmental delay versus typically developing children. Furthermore, the external validity of the Q-CHAT towards measures of cognitive functioning, language, behavior, and autism symptom severity were analyzed.

Similarly to previous studies using the Q-CHAT in both clinical and population-based settings (26–28), we found a normal distribution of the Q-CHAT scores. This result confirms the unique potential of this instrument as a dimensional measure of autistic traits along a continuum in the population and makes the Q-CHAT a particularly suitable tool to be used in genetic and biomarker stratification approaches at a very early developmental stage. As expected and consistent with the findings reported by Allison et al. (20), children with a diagnosis of autism scored significantly higher than those with typical development. Furthermore, in our study, we explored the Q-CHAT score distribution in children with developmental delay (DD) and found that Q-CHAT scores in autism children were significantly higher than those reported in DD children. Conversely, scores on the QCHAT in the DD group, after controlling for PDQ and age, were slightly higher but not significantly different from TD children. Furthermore, while an autism condition strongly predicted the Q-CHAT score, a DD condition did not. These results are worthy of attention, in that the Q-CHAT has been specifically designed as a quantitative measure for autism rather than a broadband tool for neurodevelopmental conditions (including autism) in general. Consequently, it may be expected that the Q-CHAT would be less accurate in identifying children with a DD diagnosis than those with an autism diagnosis. This was in fact the case. Children with DD were not classified consistently by the Q-CHAT (AUC = 75% indicating a modest accuracy), while the discriminant validity of the Q-CHAT for autism was very good (AUC = 89%) and in line with that reported by Allison et al. (21) (AUC = 92%). Unlike the previous findings where an effect of gender (with boys scoring higher in the unselected sample) and age (a small negative correlation in the autism group) on the Q-CHAT scores were reported (20), we did not replicate these results. Also, the mean Q-CHAT scores in boys and girls in Allison et al.’s study were somewhat higher [mean score of 27.5 (7.8) for boys and of 25.8 (7.7) for girls] than our sample [TD males = 21.6 (7.6); TD females = 20.8 (5.7)]. However, it should be considered that in Allison et al.’s study, the Q-CHAT questionnaires were sent by post and no direct assessment was possible to exclude potential children with atypical development and/or mild neurodevelopmental conditions. In our study, all the TD children were tested for language and performance development using the Griffiths test as well as for behavior using the CBCL 1.5-5, and indeed, three children (2.3%) were excluded from the study because of language/developmental delay or autism traits. The same scoring pattern has been found in the autism group with Allison et al.’s sample reporting rather higher Q-CHAT scores [mean score of 51.3 (SD = 14.1) for boys and of 54.6 (SD = 14.9) for girls] than our sample. Again, it is likely that the sample characteristics in the two studies are different in that the autism children in our study have been referred and diagnosed within clinical facilities, whilst in Allison et al.’s study the autism sample was mainly recruited through the Autism Research Centre website and parents who volunteered might have had more impaired children and/or over-reported symptoms. Also in Allison et al.’s study, neither independent verification of an autism diagnosis nor IQ assessment was possible. As for age, the unselected group in Allison et al.’s study was young [mean age (SD) = 21.2 (2.1) months], whereas children in the autism group were significantly older [mean age of 44.5 (10.2) months]. In our study, the autism and TD samples were more consistently matched [mean age (SD) = 31.6 (8) months and mean age (SD) = 33.2 (9.3) months in the autism and TD group, respectively] and an effort has been made to recruit autism children as young as possible, before the age of 3 years, to comply with the purpose of the instrument as an early screener for autism. When the Q-CHAT total score that better differentiated between autism and TD children was explored, we found that a cut-off of 27 maximized sensitivity (83%) without compromising specificity too much (78%). In a previous study, using a short version of the Q-CHAT (Q-CHAT-10), Allison et al. (21) reported a higher sensitivity and specificity (91% and 89%, respectively) at the screening cut-point. However, it should be considered that the Q-CHAT-10 included selectively only the 10 most discriminating items, and therefore, higher sensitivity and specificity may be expected. In another study, in a community clinical sample, Charman et al. (29) explored the accuracy of two other commonly used screeners, the MCHAT and the SCQ, in predicting autism versus non-autism status. While the M-CHAT demonstrated adequate sensitivity (84%) but poor specificity (50%), the SCQ conversely demonstrated low sensitivity (64%) and moderate specificity (75%). Overall, the Q-CHAT in our sample replicated the good sensitivity of the M-CHAT whilst maintaining a sub-optimal but still higher specificity than the SCQ. When an autism versus a DD status was contrasted, a higher cut-point of 31 was the most appropriate in our sample to better discriminate between the two conditions, still ensuring adequate sensitivity (73%) and specificity (76%). The latter cutoff, although not reaching the recommended sensitivity and specificity of at least 80% (30), nevertheless is still acceptable, especially considering that when there is a greater overlapping of scores, such as in the case of autism and DD, sensitivity and specificity are consequently lower. In the light of these results, two different cut-points (27 and 31, respectively) may be proposed, depending on whether the Q-CHAT is intended to be used as a broader first-level screener or more specifically used to discriminate between autism and other developmental conditions. Finally, we explored the external validity of the QCHAT with regard to measures of cognitive functioning, language, autism symptom severity, and behaviors. In the autism group, we found that Q-CHAT scores were positively correlated with the severity of symptoms in the *Social Affect* domain of the ADOS-2 and negatively correlated with the language abilities on the Griffiths test. These findings indicated that the lower the language and social communication skills, the higher the Q-CHAT scores were. Furthermore, both in autism and TD children, the Q-CHAT scores were positively correlated, with medium to large effect sizes in both groups, with the CBCL PDD subscale, as well as with the internalizing subscale (in particular emotional reactivity and withdrawn) and the externalizing subscale (attention and oppositional-defiant problems in particular). These findings are consistent with those reported by Magiati et al. (27) in a large population-based sample using the Q-CHAT and by Constantino et al. (31) and Duku et al. (32) in two independent samples of children with a diagnosis of autism using the Social Responsiveness Scale.

There are limitations to this study that must be acknowledged. First of all, there are unequal proportions of children in the three groups, with the DD group having half the sample size of the autism and TD groups.

Furthermore, DD children in our sample were significantly younger than children in the other two groups. Although age did not predict Q-CHAT scores and we controlled statistically for age, a replication in a larger and better age-matched sample of children with DD is recommended. In addition, the PDQ in TD children was high and maybe not be a representative of the general population. Nevertheless, the effect of PDQ was controlled for in all the analyses, and the results were confirmed.

While these factors have been controlled for statistically, in the application of the QCHAT in clinical and community settings, we should consider their possible effects with respect to the cut-off while deciding “caseness.”

## Conclusions

In conclusion, we demonstrated that in a clinical setting of children already diagnosed with an ASD or developmental delays as compared to typically developing children, the Q-CHAT is a quantitative, normally distributed measure with satisfying psychometric properties and external validity, able to discriminate autism children not only from children with typical development but also from children with other developmental conditions such as developmental delay. Future research should aim to replicate the findings in clinical samples from a larger community as well as in population samples with follow-up prospective designs before recommending the Q-CHAT as a clinical instrument for early autism screening.

## Ethics Statement

The protocol was approved by the Scientific Foundation “Stella Maris’ Ethic Committee” (Prot. n. 11/2012) and a written informed consent in accordance with the Declaration of Helsinki was obtained from all subjects.

## Author Contributions

LR conceived of the study, participated in its design and coordination, and drafted the manuscript. FM, SB, and CA participated in the design and interpretation of the data. GA, FA, and AG participated in the design and coordination of the study. EL, NT, CC, RM, NC, and VC performed the measurement. FC participated in the design of the study and performed the statistical analysis. GP and GT participated in the coordination of the study and helped to draft the manuscript. All authors read and approved the final manuscript.

## Funding

This research (“Toddlers Project”) was supported by the Italian Ministry of Health and Tuscany Region (GR-2010-2319668). CA and SBC were supported by the Autism Research Trust and the MRC during the period of this work. The research was supported by the National Institute for Health Research (NIHR) Collaboration for Leadership in Applied Health Research and Care East of England at Cambridgeshire and Peterborough NHS Foundation Trust. The views expressed are those of the author(s) and not necessarily those of the NHS, the NIHR, or the Department of Health.

## Conflict of Interest Statement

The authors declare that the research was conducted in the absence of any commercial or financial relationships that could be construed as a potential conflict of interest.
